# Outcomes post Ozaki procedure among children with aortic valve disease at Jakaya Kikwete Cardiac Institute, Dar es Salaam, Tanzania: a retrospective descriptive study

**DOI:** 10.1186/s12872-024-03829-8

**Published:** 2024-03-19

**Authors:** Zawadi Edward Kalezi, Alphonce Nsabi Simbila, Stella Mongella, Deogratias Nkya, Godwin Sharau, Felix Shonyela, Vivienne Mlawi, Naizihijwa Majani

**Affiliations:** 1 Department of Paediatric cardiology, Jakaya Kikwete Cardiac Institute, P.O Box 65141, Dar es Salaam, Tanzania; 2 Department of Paediatrics and Child Health, Muhimbili University of Health and Allied Sciences, Dar es Salaam, Tanzania; 3 Emergency Medicine Department, Muhimbili University of Health and Allied Sciences, Dar es Salaam, Tanzania

**Keywords:** Ozaki procedure, Aortic valve, Paediatrics, Tanzania

## Abstract

**Background:**

Aortic valve reconstruction using glutaraldehyde-treated autologous pericardium, also called Ozaki procedure, is a surgical procedure for patients with aortic valve disease. Gratifying results have been reported in adult patients, however, limited published data is available in paediatric population. This study looked at clinical characteristics and early outcomes of children who underwent Ozaki procedure at our Institute.

**Methods:**

This was a retrospective descriptive study conducted on children who underwent aortic valve reconstruction at Jakaya Kikwete Cardiac Institute (JKCI) from January 2019 through December 2022. Medical records of these children were reviewed to extract data on demographics, clinical characteristics, redo surgical interventions and survival.

**Results:**

A total of 10 children underwent Ozaki procedure during the study period. Eight children had severe aortic regurgitation while 2 had severe aortic stenosis preoperatively. All children had either none or trivial aortic regurgitation immediately after surgery. None of them had redone operations throughout the follow-up period. There was no in-hospital mortality, however, one child died one-year after surgery. The mean follow-up period was 1.6 years with the longest follow-up time of 4 years.

**Conclusion:**

Ozaki procedure showed encouraging early results among children with aortic valve disease who underwent surgical repair by this technique. Future studies with larger sample sizes and longer follow up periods to evaluate long-term results in this population are recommended.

## Background

Aortic valve (AV) diseases can be classified as congenital or acquired. Acquired aetiologies of aortic valve diseases include post rheumatic fever sequela resulting into rheumatic heart disease (RHD), those secondary to infective endocarditis, those due to connective tissue disorders, or due to degenerative atherosclerotic process [1, 2]. RHD is reported to be the most predominant acquired heart condition in northern Tanzania contributing to 50% of children with acquired heart diseases. Close to 8% has been found to have isolated aortic valve regurgitation [3]. Similar to what has been reported in India [4].

Surgical options for children with aortic valve disease can either be repair or replacement, depending on the time of presentation and ventricular dysfunction [2]. In the past, most aortic valves were being replaced but due to complexity of some procedures (Ross procedure) and lack of availability, currently there is increasing surgical experience for aortic valve repair [1, 2, 5]. Talwar et al. reported cuspal thinning and commissurotomy being mostly done with gratifying long-term outcomes [6]. Bacha and his colleagues reported similar findings, however, more than two-third of those with repaired aortic valve needed re-interventions [7].

Over a decade ago, Shigeyuki Ozaki proposed a procedure which consists of glutaraldehyde-treated autologous pericardium to replace aortic valve leaflets. Since no foreign material is required, the procedure is considered as a repair rather than a replacement [6, 8]. Ozaki procedure can be applied to a wide spectrum of AV diseases including aortic regurgitation or stenosis, infective endocarditis, and prosthetic valve endocarditis [1]. Apart from low reoperation rates, avoidance of the need for lifelong anticoagulation therapy [8] after the procedure resonates well with economic conditions of our patients especially in the low- and middle- income countries (LMIC).

A 16-years follow-up study post aortic valve reconstruction using a single strip autologous pericardium reported good outcomes in terms of survival but the freedom from reoperation was below 50% [9]. On the other hand, Ozaki and colleagues described good outcomes post aortic valve reconstruction using the Ozaki technique. The freedom from death and reoperation of almost 10 years follow-up time, were reported to be 85.9% and 96.2% respectively [8]. Similar results were reported by Wiggins and colleagues in London and Polito et al. [10, 11].

The Ozaki technique has promising results in paediatric population despite paucity of published data. With limited availability of paediatric cardiac services in LMICs Ozaki procedure seems to be a good option. This study therefore aimed at documenting the short-term clinical results of the children who underwent Ozaki procedure at our institute, as well as their survival.

## Methods

### Study design and setting

This was a hospital based retrospective descriptive study conducted at the Jakaya Kikwete Cardiac Institute (JKCI), Dar es Salaam, Tanzania from January 2019 to December 2022. JKCI is a 150-bed capacity national referral and teaching centre which provides specialized cardiac services for both adults and children. It is the only centre offering open cardiac surgery in Tanzania with a dedicated paediatric operating theatre and two paediatric cardiac surgeons.

### Study participants and variables

Medical records of all children with aortic valve disease who underwent Ozaki procedure at JKCI within 4 years were reviewed, however, those with incomplete data were excluded. Clinical parameters were age, sex, date of admission, preoperative valvular diagnosis, preoperative and postoperative echocardiographic details (peak and mean pressure gradients across aortic valve, jet length, peak velocity, and valve type), previous operations, date of surgery, type of cardiac surgery, and date of discharge. The survival and re-do operations were determined immediately post-surgery, over hospital stay and post discharge.

### Surgical technique

The Ozaki surgical technique involved harvesting autologous pericardium first after routine median sternotomy. The preparation started by cleaning fat and other redundant tissue on the outer surface of the autologous pericardium. The pericardium was excised and then treated with 0.6% glutaraldehyde solution for 10 min. The treated pericardium was rinsed using physiologic saline solution. Then, the distance between each commissure measured using a special measuring device. The new cusp with the size corresponding to the measured value was trimmed with an original template from glutaraldehyde treated autologous pericardium. The annular margin of the pericardial cusp was sutured along each annulus. The smooth (inner) surface of pericardium was placed on the left ventricular side. The pericardial cusp was then sewn thoroughly up to the top of commissure. The function of the substituted valves was assessed by transesophageal echocardiography (TEE).

### Data management and analysis

Data entry and cleaning was done using Statistical Package for Social Science (SPSS) version 25. Description of means, medians, frequencies, and proportions of the given data of each variable were calculated. Contingency tables were constructed for bivariate analysis. For the missing data, case deletion approach was used.

Ethical approval to conduct the study was obtained from the Ethics Review Committee of the Jakaya Kikwete Cardiac Institute (Ref.No.AB.123/307/38).

## Results

**Fig. 1:**
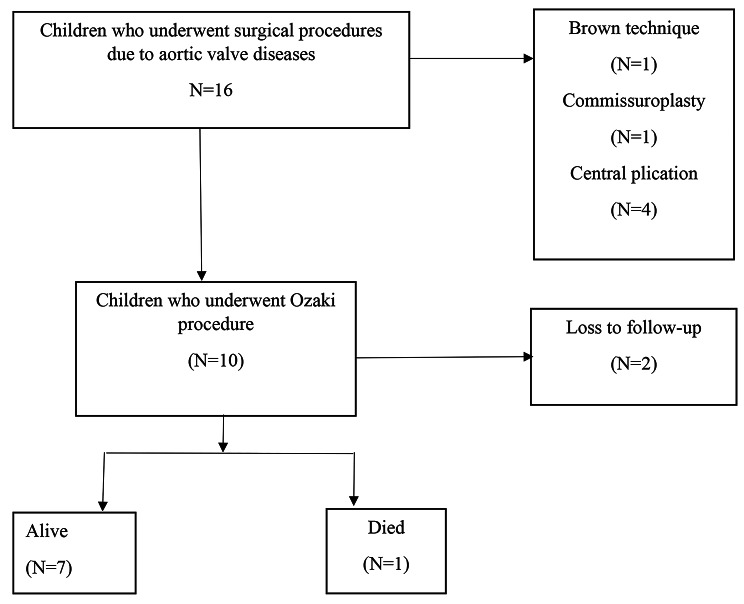
Flow diagram showing recruitment of the study participants and their outcomes

A total of 16 patients underwent surgical procedures due to aortic valve disease during the study period. Ten children had acquired heart disease while six were congenital. Ten children met the inclusion criteria and were enrolled into the study (Fig. 1).

Ten children underwent aortic valve reconstruction (Ozaki procedure) during the study period, of whom eight were aged 10 years and above. There were seven patients who underwent concomitant procedures: Mitral valve repair and replacement, *n* = 2 and *n* = 1 respectively; aortic aneurysm repair, *n* = 2; vegetation removal, *n* = 1; defect closure, *n* = 1. One patient had bicuspid aortic valve while nine had tricuspid morphology (Table 1).

**Table 1:** Baseline patients’ characteristics, *N* = 10

VARIABLE	FREQUENCY(*N*)
**AGE (YEARS)**	
< 10	2
10–19	8
**SEX**	
Male	7
Female	3
**SURGERY INDICATION**	
Aortic regurgitation	7
Aortic stenosis	2
Infective endocarditis	1
**CONCOMITANT SURGERY**	
Mitral valve replacement	1
Mitral valve repair	2
Aortic aneurysm repair	2
Defect closure surgery	1
Vegetation removal (Infective endocarditis)	1
**MORPHOLOGY OF AORTIC VALVE**	
Bicuspid	1
Tricuspid	9

Seven out of 10 children had rheumatic heart disease, two had congenital aortic stenosis together with aortic aneurysms secondary to infective endocarditis. Most of the participants had either none or trivial aortic regurgitation immediately post-surgery (Table 2).

**Table 2:** Echocardiographic parameters and outcomes post Ozaki procedure

Patient	Age (yrs)	Pre-operative Diagnosis	Pre and post operative aortic valve findings	Follow-up time	Neo-aortic valve findings	Reoperation	Survival
1	16	Rheumatic heart disease	Severe AR/ No AR	4	No AR	None	Alive
2	17	Congenital Aortic stenosis with aortic cusp vegetations	Severe AS/ No AS, trivial AR	1	No AS, Mild AR	None	Died
3	9	Rheumatic heart disease	Severe AR/ Trivial AR	2	Mild AR	None	Alive
4	12	Rheumatic heart disease	Severe AR/ No AR	2	Trivial AR	None	Alive
5	16	Rheumatic heart disease	Severe AR/ No AR	1	No AR	None	Alive
6	14	Rheumatic heart disease	Severe AR/ No AR	1	Mild AR	None	Alive
7	14	Rheumatic heart disease	Severe AR/ Trivial AR	1	Trivial AR	None	Alive
8	9	Rheumatic heart disease	Severe AR/ No AR	3	No AR	None	Alive

AR; Aortic regurgitation, AS; Aortic stenosis

Out of ten study participants, seven children were alive, and two patients were lost to follow- up. One death was that of a 17-year-old with congenital aortic stenosis and aortic cusp vegetations who underwent Ozaki procedure for severe aortic regurgitation. He died of unknown cause in peripheral hospital one-year post surgery. None of them had redone interventions throughout the follow-up period. The mean follow-up time was 1.6 years with the longest follow-up time of 4 years. (Table 2).

## Discussion

In the past, aortic valves were mostly replaced but recently there are variety of options for aortic valve reconstruction. This study aimed to determine echocardiographic haemodynamic parameters and survival of the children with aortic valve disease who underwent Ozaki procedure at our Institute for the past 4 years. Out of 16 children who underwent surgery due to aortic valve disease, ten had aortic valve reconstruction by Ozaki technique. Among them only one child died.

In this study, all the children had either none or trivial residual valve regurgitation from severe form preoperatively. This is because, aortic valve reconstruction using the Ozaki technique makes the coaptation zone longer than the native valve, hence reducing postoperative aortic insufficiency. Wiggins and colleagues echoed the above outcomes by reporting 100% freedom from moderate to severe AR post Ozaki procedure during follow-up [10]. Additionally, none of our study participants had a reported redo surgery throughout the follow-up period. Similar findings were also reported in India and Japan [4, 8].

Among those who underwent aortic valve reconstruction, aortic valve pathology was mostly a result of rheumatic heart disease. This is due to the fact that rheumatic heart disease is much prevalent in resource limited countries including Tanzania as reported by Allen and his colleagues [2]. Conversely, the father of the Ozaki technique reported degenerative calcification in the majority of his patients which was due to comparative age difference [8].

Despite the two patients who were lost to follow up, seven children were alive during the follow-up period. Only one child died at a peripheral hospital a year later, however, the exact cause of death was uncertain. The above can be due to few reported valve-related complications including endocarditis following reconstructive surgery as published by Ozaki and his colleagues as well as Aicher et al. [8, 12, 13]. Apart from endocarditis, valve calcification has been reported though different material other than autologous pericardium were used [10, 14].

In addition to low reoperation rates [8], there is no need for lifelong anticoagulation after Ozaki procedure which resonates well with economic conditions of our patients. Most of our patients have financial constraints and availability of warfarin in our country is only promising at hospital level, hence, this procedure does not only avoid complications following long-term use but is also cost-effective [8, 12]. It reduces the financial burden generated by direct and indirect healthcare costs including transport to and from the hospital especially those in remote areas. On the other hand, size mismatch between protheses and our growing children make early-redo surgical interventions unavoidable.

The limitations of our current study are attributed to its retrospective nature, and it is from a single institution. Additionally, a small number of children underwent this procedure. This is due to the fact that aortic valve in children is not as commonly affected as mitral valve in view of RHD though children are presenting at advance stage requiring surgical intervention.

## Conclusion

Aortic valve reconstruction using Ozaki technique has shown encouraging early outcomes despite limitations, this is a wakeup call for more future studies with longer follow up time for long-term results.

## Data Availability

Datasets used during the study are available from the corresponding author upon reasonable request.

## Abbreviations

AR: Aortic regurgitation
AS: Aortic stenosis
AV: Aortic valve
ECHO: Echocardiography
JKCI: Jakaya Kikwete Cardiac Institute
RHD: Rheumatic heart disease
TEE: Trans esophageal echocardiography
VSD: Ventricular septal defect
